# *Salmonella* Typhi genotypic diversity, cluster identification and antimicrobial resistance determinants in Mukuru settlement, Nairobi Kenya

**DOI:** 10.1186/s12879-024-09635-z

**Published:** 2024-07-24

**Authors:** Purity Kasiano, Masatomo Morita, Toshio Kodama, Hirotaka Hiyoshi, Susan Kavai, Susan Kiiru, Samuel Kariuki

**Affiliations:** 1 Centre for Microbiology Research, Kenya Medical Research Institute, Nairobi, Kenya; 2 Jomo Kenyatta University of Agriculture and Technology, JKUAT, Nairobi, Kenya; 3 Department of Bacteriology I, National Institute for Infectious Diseases, Tokyo, Japan; 4 Department of Bacteriology, Institute of Tropical Medicine, Nagasaki University, Nagasaki, Japan; 5 DEJIMA Infectious Disease Research Alliance, Nagasaki University, Nagasaki, Japan; 6 Drugs for Neglected Diseases initiative, Eastern Africa, Nairobi, Kenya

**Keywords:** *Salmonella* Typhi, Genotype, Antimicrobial Resistance

## Abstract

**Background:**

Understanding the source of typhoid infections and the genetic relatedness of *Salmonella* Typhi (*S*. Typhi) by cluster identification in endemic settings is critical for establishing coordinated public health responses for typhoid fever management. This study investigated the genotypic diversity, antibiotic resistance mechanisms, and clustering of 35 *S.*Typhi strains isolated from cases and carriers in the Mukuru Informal Settlement.

**Methods:**

We studied 35 *S*.Typhi isolates, including 32 from cases and 3 from carriers, from study participants in the informal settlement of Mukuru, Nairobi, Kenya. Genomic DNA was extracted, and whole-genome sequencing (WGS) was performed to determine the phylogenetic relatedness of strains and detect antimicrobial resistance determinants (AMR). WGS data were analyzed using bioinformatics tools available at the Center for Genomic Epidemiology and Pathogenwatch platforms.

**Results:**

Genotype 4.3.1.2 EA3 was found to be dominant at 46% (16/35), followed by 4.3.1.2 EA2 at 28% (10/35), and 4.3.1.1 EA1 at 27% (9/35). A comparison of the isolates with global strains from Pathogenwatch identified close clustering with strains from Uganda, Tanzania, Rwanda, and India. Three isolates (9%) distributed in each cluster were isolated from carriers. All genotype 4.3.1.2 EA3 isolates were genotypically multidrug-resistant to ampicillin, chloramphenicol, and trimethoprim-sulfamethoxazole. Single mutations in the quinolone resistance-determining region were identified in the *gyrA* (S83Y) and *gyrB* (S464F) genes. All isolates associated with multidrug resistance showed the presence of the IncQ1 plasmid with the following genes: *blaTEM-1B*,* catA1*,* sul1*,* sul2*, and *dfrA7*.

**Conclusion:**

The close phylogenetic relatedness between antimicrobial-resistant case isolates and carriage isolates indicates that typhoid carriage is a possible source of infection in the community. Comparative analysis with global isolates revealed that the Kenyan isolates share common lineages with strains from neighboring East African countries and India, suggesting regional dissemination of specific MDR clones. AMR was a major feature of the isolates. Surveillance and testing for antimicrobial susceptibility should inform options for the management of cases.

**Supplementary Information:**

The online version contains supplementary material available at 10.1186/s12879-024-09635-z.

## Background

*Salmonella enterica* subspecies *enterica* serovar Typhi is the causative agent of typhoid fever, a disease endemic to overpopulated urban areas, characterized by overcrowding, poor sewage systems, and poor hygiene and sanitation standards [1]. Typhoid fever causes approximately 26.9 million cases and 217,000 deaths yearly, mainly affecting children and the elderly [2]. *S.*Typhi is a human-restricted pathogen that persists in a population through asymptomatic individuals who develop a carrier state post-infection [3].

In Kenya, typhoid fever remains a significant public health challenge [4]. Studies such as Ng’eno et al. (2023) have explored the disease burden in Kibera, an urban settlement, over 10 years (2010–2019) and reported a resurgence of typhoid fever in 2019 after a prolonged period of low rates, with the highest incidence among children and a high multidrug-resistant (MDR) prevalence of 71.9% [5]. Other studies from Kenya have also reported on the high prevalence of MDR *S*.Typhi strains [1, 6]. These findings highlight the need for ongoing surveillance and targeted interventions. Antibiotic treatment for both asymptomatic carriers and acute cases has contributed to the emergence of MDR strains of *S*.Typhi, significantly complicating treatment options [7]. This has been attributed to the misuse and overuse of antibiotics for example the inappropriate selection of antibiotics, incorrect dosages, and incomplete treatment courses, which contribute to the selection pressure that drives the development of multidrug-resistant (MDR) strains [8]. Although vaccines for typhoid are available, their coverage in Kenya and the surrounding region remains limited [9].

Notably, genotype 4.3.1 (formerly haplotype H58) has been closely associated with MDR traits [10]. Originally from Southeast Asia, genotype 4.3.1 has spread globally over the past 30 years, becoming prevalent in regions including Kenya, Tanzania, Malawi, and Southern Africa [3, 11]. The spread of genotype 4.3.1, which displaces antibiotic-sensitive strains, is attributed to selective pressures from regional antibiotic use [12]. Presently, genotype 4.3.1 is subdivided into three lineages 4.3.1.1, 4.3.1.2, and 4.3.1.3 with further subdivision on lineage I and II to monitor East African variants which include sub-lineages EA1, EA2, EA3 [13].

In Kenya, the dominance of the MDR genotype 4.3.1 strains is well-documented. For example, Kariuki et al. (2021), examined the dynamics of typhoid infection and carriage among children < 16 years [10]. Similar findings by other authors in Kenya, specifically from studies conducted in the informal settlement of Kibera underline the evolutionary success of genotype 4.3.1 [14].

Clusters of *Salmonella*, representing highly genetically related isolates from a common source, are critical for identifying outbreaks and guiding epidemiological interventions [11]. This study aims to investigate the clusters, genotypic diversity, and antimicrobial resistance mechanisms of 35 *S*.Typhi isolates from cases and carriers in the Mukuru Informal Settlement, previously identified by serotyping and archived by Kasiano et al. (2024) [6]. By performing WGS in the current study, we aim to achieve a more detailed understanding of the genetic diversity, phylogenetic relationships, and resistance mechanisms of the *S*.Typhi isolates. Additionally, it seeks to conduct a comparative analysis of these isolates with global *S.*Typhi strains to understand their distribution and relatedness.

## Materials and methods

### Study area

Mukuru Settlement is one of Nairobi’s largest and most crowded urban slums and is home to over 250,000 people [6]. As an informal settlement, there is a shortage of safe drinking water and inadequate infrastructure and sanitation, which predisposes people to typhoid fever [1]. Moreover, patients have easy access to over-the-counter medications, which fuels the growth and spread of antibiotic resistance [15]. For instance, Kavai et al. (2018) reported an MDR prevalence of 55.5% in *S.*Typhi isolates from outpatient clinical samples from Mukuru villages in Nairobi [1]. To effectively develop typhoid control and prevention methods, it is important to understand the epidemiological dynamics of *Salmonella* Typhi in this setting.

### Sample collection

The isolates used in this study were obtained from samples archived during our previous investigation collected from July 2021 to July 2022. Sampling was done as described in Kasiano et al., 2024 [6]. Briefly, participants were recruited from three health centers within the Mukuru settlement area: Medical Missionaries of Mary (MMM), Municipal County Council Clinic (MCC), Mukuru Kwa Reuben Clinic (MR), and an inpatient facility, Mama Lucy Kibaki Hospital (MLK) which is situated in Embakasi constituency and serves as a reference facility for the Mukuru area.

Isolates were obtained from 1014 study participants, with the majority presenting with typhoid-like symptoms. The inclusion criteria were patients presenting with a temperature of ≥ 37.5 °C and a history of fever lasting more than 3 days. Carrier sampling involved collecting stool samples from individuals residing in the same households as confirmed typhoid cases. This was done to investigate whether there was any genetic similarity or relationship between strains obtained from carriers and cases and to examine whether the carriers were the source of typhoid fever in the area.

In our earlier study, *S*.Typhi confirmation involved culture and serotyping techniques [6]. After a patient was confirmed to have *S*.Typhi at least one contact living with the case was requested to provide a stool sample to check for carriage.

### DNA extraction

Archived *S.*Typhi isolates were stored in glycerol at -70 °C and were sub-cultured and revived for DNA extraction. DNA isolation was done using the GenElute bacterial DNA kit (Sigma-Aldrich), and the concentrations were measured using Nanodrop (Thermo Fisher Scientific) and stored at -80℃. DNA was shipped according to the Kenya Medical Research Institute material transfer agreement guidelines (MTA) to the Department of Bacteriology I, National Institute of Infectious Diseases, Japan, for whole-genome sequencing.

#### Whole-genome sequencing

Before genomic library preparation, the genomic DNA concentration was measured using the Qubit 4.0 fluorometer (Thermo Fisher Scientific). Following the manufacturer’s instructions, a genomic library was prepared using the QIAseq FX DNA Library kit (Qiagen), and an equal amount of each library was pooled. The pooled libraries were size selected on a BluePippin (Sage Science) and then quantified using the Kapa Library Quantification Kits for Illumina platforms (Roche). Sequencing was performed on a MiSeq with the MiSeq Reagent Kit v3 (600-cycle) (Illumina).

### Data analysis

We used the “fastp” with default parameters for data QC https://github.com/OpenGene/fastp. Genome assembly was performed using SPAdes v.3.13.0 with the “--careful” and “--cov-cutoff auto” options [16] after processing the raw reads with fastp v.0.20.1 [17] and contigs less than 500 bp in length were removed from the draft genome. CheckM (version 1.1.6) was used to assess the quality and check for contamination of the draft genome.

The draft genomes were analyzed using online bioinformatics pipelines at the Center for Genomic Epidemiology (CGE) of the Technical University of Denmark (http://www.genomicepidemiology.org/services/*).* Species identification was performed using PathogenWatch (https://pathogen.watch/*).* This involved uploading draft genomes to the web-based platform, which contains a database of reference genomes for accurate identification of the pathogens. Genotyping of the isolates (as called by single nucleotide polymorphism) was performed using the GenoTyphi scheme [12] at the PathogenWatch platform (https://pathogen.watch/*).* For confirmation, *Salmonella* serovars were also predicted using SeqSero v1.2 (https://cge.food.dtu.dk/services/SeqSero/*)* [18]. The determination of the MLST profile was performed using MLST v2.0 (https://cge.food.dtu.dk/services/MLST/*)* [19]. Identification of resistance genes was done using ResFinder v4.4.3 (https://cge.food.dtu.dk/services/ResFinder/*)* [20]. Plasmid presence was investigated using PlasmidFinder v2.1 (https://cge.food.dtu.dk/services/PlasmidFinder/*)* [21, 22].

To analyze the phylogeny and genetic relatedness of the isolates, high-quality paired-end reads were aligned to the reference genome *Salmonella* Typhi Ty2 to obtain single nucleotide polymorphisms (SNPs) that formed a basis of the phylogenetic inference. Maximum Likelihood (ML), specifically the RAxML tool was used to construct the phylogenetic tree. Recombinant regions in the sequences were filtered to ensure the phylogeny reflected vertical genetic inheritance and a Newick format (.nwk) phylogenetic tree file was generated. The tree was further visualized using the Microreact platform incorporating metadata associated with the samples [23].

We further investigated the phylogenetic relatedness and diversity of our *S*.Typhi against other strains in the Pathogen platform to provide insights into their phylogeographical distribution. This was done by forming a collection with isolates from Uganda, Tanzania, South Africa, Rwanda, and India. A single nucleotide polymorphism (SNP) tree was generated directly from the platform and the resulting Newick(.nwk) tree file was downloaded. The tree file was then uploaded and visualized on the Microreact platform.

## Results

### Genotypic diversity and phylogeographical relatedness

Among the 35 *S.*Typhi isolates confirmed by WGS, 32 were from cases, and an additional 3 isolates were obtained from carriers identified through contact tracing and household sampling.

**Fig. 1:**
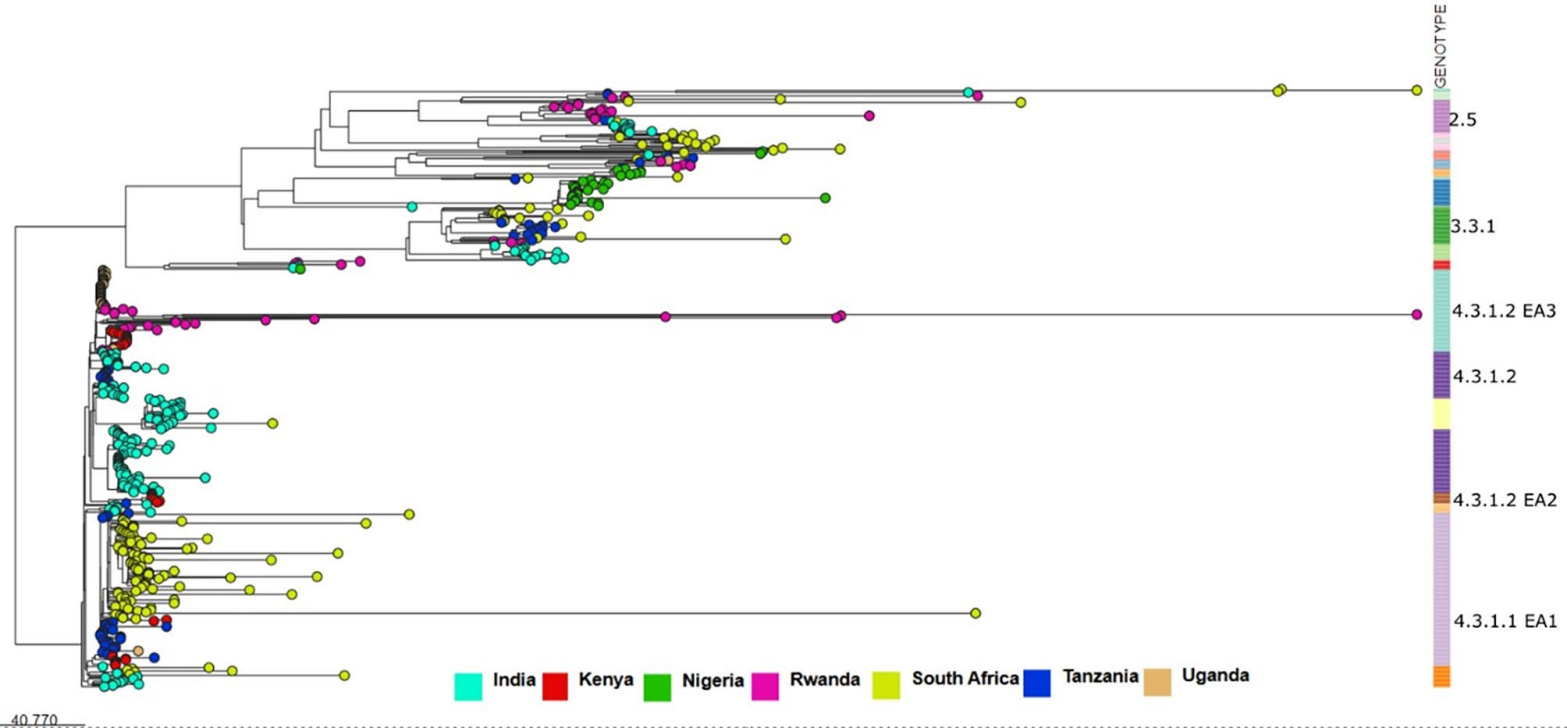
Showing the comparison between the Kenyan isolates used in this study and the global data from Pathogenwatch. The phylogenetic tree shows a close clustering of Kenyan isolates with isolates from Tanzania, Uganda, Rwanda, and India

We incorporated a total of 504 global *S*.Typhi isolates (1991–2019) from Pathogenwatch and formed a collection https://pathogen.watch/collection/xaj3i7y7uw8v-snppk. These included isolates from Uganda, Rwanda, Tanzania, Nigeria, South Africa and India. A SNP tree was constructed on the Pathogenwatch platform, and the tree was visualized using Microreact https://microreact.org/project/8PDD6WLcHAUt3KEDctTFno-snppk. (Fig. 1). This phylogenetic tree provides insight into the genetic diversity and evolutionary history and places the 35 Kenyan isolates from this study within a broader context to enhance the understanding of regional and global transmission patterns.

As observed on the tree, the Kenyan isolates are mapped to known global clades and are distributed across three distinct clusters on the phylogenetic tree, indicating multiple introduction events. This may imply that different strains of *S*.Typhi were introduced independently in the country. The 35 isolates show clustering and connections with isolates from Uganda, Rwanda, Tanzania, and India suggesting both local and cross-border transmissions.

In one of the clades, Kenyan and Tanzanian isolates are in proximity suggesting ongoing genetic exchange in the two bordering countries. Similarly, the two other clades with Kenyan isolates show a close clustering of Rwanda, India, and Ugandan isolates which may imply a possible shared evolutionary history and potential routes of transmission between these regions through interactions in the form of trade and travel (Fig. 1).

### Phylogenetic relatedness of the isolates

**Fig. 2:**
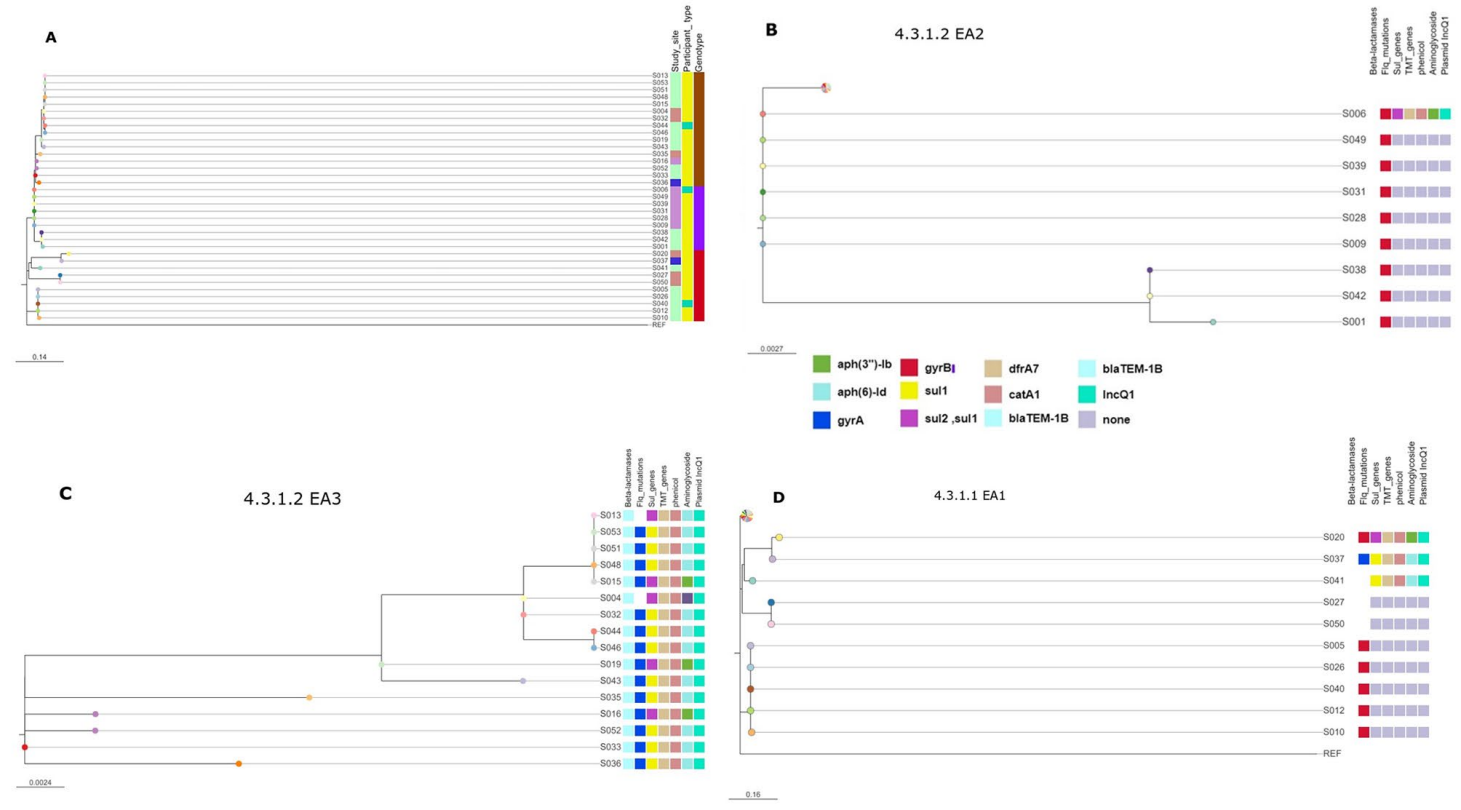
Phylogenetic Tree of *Salmonella* Typhi strains Isolated from Mukuru Settlement, Nairobi, Kenya. **A**-The main phylogenetic tree visualized using Microreact, revealing distinct clusters of isolates and genotypes, suggesting localized transmission events within the settlement. (**B**–**D**) Represent the different genotypes collapsed from the main tree and viewed separately and their corresponding antimicrobial determinants. The colored leaf nodes at the end of the branches represent the id of the isolates

All 35 *S.*Typhi isolates were sequence type 1 (ST1), as identified by MLST profiling. The strains were highly diverse and classified into genotypes 4.3.1.1 EA1, 4.3.1.2 EA2, and 4.3.1.2 EA3 (Fig. 2A) where 4.3.1.2 EA3 was found at a high frequency of 46% (16/35), followed by 4.3.1.2 EA2 at 28% (10/35), and 4.3.1.1 EA1 at 27% (9/35).

The isolates clustered in 3 main clades represented by their genotypic lineages. Clade 1 (Fig. 2B) consisted of 9 isolates. The isolates in this clade were closely linked with a series of short branches suggesting minimum genetic divergence and a common recent ancestor.

Clade 2 (Fig. 2C) was the largest among the three clades consisting of 16 isolates. The isolates in this clade showed genetic diversity as shown by the longer branches in the clade and distinct sub-clades. This suggests that some isolates may have undergone more genetic changes compared to others over time. Isolates S35, S16, S52, S33, and S36 showed divergence from the other two sub-clades.

Clade 3 (Fig. 2D) consisted of 10 isolates. The isolates in this group showed a close similarity as indicated by the short branches separating most of the isolates. This suggests that the isolates shared a recent common ancestor. However, despite the close relationship, there is evidence of slight genetic diversity within this clade among isolates S020, S037, S041, S027, and S050.

### Antimicrobial resistance determinants

The following genes associated with antimicrobial resistance were detected: *bla-*_*TEM1B*_ at 57% (ampicillin resistance), *dfrA7* at 57%, *sul2*, *sul1* at 20%, *sul1* at 37% (resistance to trimethoprim and sulfonamides), *catA1* at 57% (chloramphenicol resistance), and *aph(6)-Id at 37%* (streptomycin resistance). Through sequence/bioinformatic analysis, these acquired resistance genes were likely associated with the plasmid IncQ1, which was detected in 57% (20/35) of the *S.*Typhi isolates. Specifically, sixteen isolates of genotype 4.3.1.2 EA3 all showed the presence of the plasmid (Fig. 2C), while three isolates of genotype 4.3.1.2 EA2 (Fig. 2B) and one isolate of 4.3.1.1 EA1 (Fig. 2D) also had the plasmid.

Resistance towards quinolones/fluoroquinolones was mediated by chromosomal mutations in the quinolone resistance-determining (QRDR) region of the DNA gyrase gene. Mutations were found in the following codons: *gyrA* (S83Y) in 49% of the isolates and *gyrB* (S464Y) in 43%. No mutations were identified in the *parC* and *parE* genes. Notably, no 3rd generation cephalosporins resistance gene determinants e.g. *bla-*_*CTX−M*,_*bla-*_*SHV*_, *bla-*_*TEM−3*,_ etc. were found in ResFinder 4.3.3 pipelines at the time of analysis.

## Discussion

All the 35 *S*.Typhi isolates confirmed by WGS in this study were found to be of Sequence Type (ST) 1 which is comparable to previous studies done in Kenya and Zambia [2, 10]. The dominance of ST1 in endemic regions has been attributed to its ability to escape innate immunity, persisting in human carriers and expression of virulence genes [24].

This study analyzes the *S*.Typhi isolates obtained from previous work by Kasiano et al. (2024), which investigated the isolation rate, carriage, and antimicrobial resistance of Typhoidal *Salmonella* in Mukuru [6]. Through WGS, we observed that all isolates were genotype 4.3.1, specifically lineages I and II, and belonged to the East African sub-lineages EA1, EA2, and EA3. However, 4.3.1.2 EA3 was the most frequent at 46% (16/35), which contrasts with the findings of Kariuki et al. (2021), who reported 4.3.1.2 EA2 as the most dominant. This variation could be attributed to the smaller sample size in our study compared to the larger sample of 240 isolates in their study.

The isolates in this study were distributed across three major phylogenetic clusters with the carrier isolates (S06, S40, S44) also distributed in each cluster. This distribution suggests that carriers may play a significant role in the transmission and endemicity of typhoid fever in informal settlements. Specifically, S40 showed close genetic relatedness to the case isolates in the 4.3.1.2 EA3 cluster, and S06 and S44 were closely related to the case isolates in the 4.3.1.2 EA2 and 4.3.1.1 EA1 cluster respectively. These findings imply that the carriers and cases share recent common ancestors, affirming the idea that carriers may contribute to the spread and persistence of *S*.Typhi in a population. This is consistent with earlier studies that investigated the significance of asymptomatic individuals in the transmission dynamics of typhoid fever in Kenya​​ [10].

All three East African sub-lineages harbored mutations for decreased ciprofloxacin susceptibility (DCS). Specifically, a *gyrA* mutation (S83Y) was present in 49% (17/35) of the isolates, and a *gyrB* mutation (S464Y) was identified in 43% (15/35) of the isolates. These findings are consistent with those of Wong et al. (2015), who reported similar mutations in the quinolone resistance-determining region (QRDR) [3]. The higher frequency of *gyrA* mutations compared to *gyrB* mutations aligns with the findings of Acheampong et al. (2019), indicating a prevalent mutation pattern in Africa where it has been documented that the prevalent mutation known to account for DCS in *S*.Typhi isolates is found in the *gyrA* gene [25]. These observations likely reflect the therapeutic use of fluoroquinolones to treat typhoid caused by multidrug-resistant (MDR) *S*.Typhi in Mukuru settlement. In addition, no mutations were observed in *parC* or *parE* genes in this study, consistent with previous studies [10]. Fluoroquinolones target the DNA gyrase subunits (*gyrA* and *gyrB*) and topoisomerase components (*parC* and *parE*). Mutations in these genes can decrease the susceptibility to fluoroquinolones and occur more frequently in genotype 4.3.1 isolates than in other *S*.Typhi haplotypes [3]. These findings emphasize the importance of antimicrobial stewardship and the development of alternative treatment strategies like vaccine administration to combat the spread of antimicrobial resistance.

The gene *bla*_*TEM−1B*_, which codes for resistance to ampicillin, was among the common resistance genes observed at 57% (20/54), similar to a study in Zambia [2]. In addition, most of the resistance determinants for phenicols and folate pathway antagonists identified in this study were similar to those identified in previous studies [26]. The identification through bioinformatic analysis, of the IncQ1 plasmid, associated with multiple antimicrobial resistance genes i.e. *catA1*,* bla*_*TEM−1B*_, *sul2*,* sul1*,* and dfrA7* genes, was observed in 20 isolates. This indicates the role of mobile genetic elements in spreading resistance genes. Similar findings have been reported in South Africa, where the IncQ1 plasmid was identified in genotype 4.3.1 *S.*Typhi isolates [11]. The identification of these resistance determinants underscores the importance of strict antimicrobial use.

A comparison of isolates from this study with the global data in PathogenWatch revealed that the Kenyan isolates are clustered closely with those of other East African (Rwanda, Tanzania, Uganda) and a South Asian country (India). This finding is in line with existing literature that genotype 4.3.1, originating from South Asia, has disseminated across different regions, establishing itself in East and Southern Africa. The phylogenetic analysis also revealed multiple introductions and local evolution of the pathogen. The close clustering of isolates from India and African countries underscores the potential routes of intercontinental pathogen transmission. This clustering indicates ongoing transmission events and regional outbreaks impelling the public health importance of adopting well-coordinated interventions to control the spread of typhoid fever.

In summary, this study gives insight into the epidemiology of typhoid fever in an endemic area by underlining the genotypic diversity, cluster identification, and antibiotic resistance determinants. Characterizing MDR strains and their genetic relatedness in this study provides preliminary information on factors that may fuel antimicrobial spread and contribute to potential outbreaks in resource-limited settings. The patterns of dissemination and persistence of MDR strains can be achieved through surveillance of resistance genes and distribution in these isolates. The results presented here come from few isolates, thus, larger extensive studies to confirm these patterns are required to be implemented. However, the presented data has significant public health implications imparting strategies to be adapted to prevent and control typhoid diseases in endemic regions. Strategies including vaccination campaigns, improved sanitary facilities, and easy access to clean water can decrease the disease burden Furthermore, evidence-based interventions and typhoid outbreak risk mitigation depend on focused surveillance programs toward carriers and active antibiotic resistance monitoring.

## Conclusion

In conclusion, typhoid fever in Mukuru is driven by the genetically diverse and consistently evolving genotype 4.3.1 *S*.Typhi. Cluster identification depicted carriers as possible primary source of typhoid infections in the community. The high prevalence of AMR determinants identified from cases warrants improved hygiene and sanitation provision including continued surveillance. This data could be helpful for targeted and timely epidemiological investigations and public health response.

### Study limitations

This study adopted a cross-sectional design and hence could not perform long-term follow-up of carriers to observe their shedding patterns and duration.

### Electronic supplementary material

Below is the link to the electronic supplementary material.

Supplementary Material 1

## Data Availability

This Whole Genome Shotgun project has been deposited at DDBJ/ENA/GenBank under the following BioProjects and accession numbers: BioProject PRJNA1064142: Accession number: JAYWIQ000000000. The version described in this paper is version JAYWIQ010000000. https://www.ncbi.nlm.nih.gov/bioproject/PRJNA1064142BioProject PRJNA1065890:Accession numbers: JAYXJA000000000 to JAYXKH000000000. The version described in this paper is version JAYXJA010000000. https://www.ncbi.nlm.nih.gov/bioproject/PRJNA1065890.
